# Multi-Depth Computer-Generated Hologram Based on Stochastic Gradient Descent Algorithm with Weighted Complex Loss Function and Masked Diffraction

**DOI:** 10.3390/mi14030605

**Published:** 2023-03-06

**Authors:** Jiale Quan, Binbin Yan, Xinzhu Sang, Chongli Zhong, Hui Li, Xiujuan Qin, Rui Xiao, Zhi Sun, Yu Dong, Huming Zhang

**Affiliations:** 1 State Key Laboratory of Information Photonics and Optical Communications, Beijing University of Posts and Telecommunications, Beijing 100876, China; 2 Beijing National Research Center for Information Science and Technology, Tsinghua University, Beijing 100084, China

**Keywords:** holographic display, mask, weighted complex loss function, gradient descent

## Abstract

In this paper, we propose a method to generate multi-depth phase-only holograms using stochastic gradient descent (SGD) algorithm with weighted complex loss function and masked multi-layer diffraction. The 3D scene can be represented by a combination of layers in different depths. In the wave propagation procedure of multiple layers in different depths, the complex amplitude of layers in different depths will gradually diffuse and produce occlusion at another layer. To solve this occlusion problem, a mask is used in the process of layers diffracting. Whether it is forward wave propagation or backward wave propagation of layers, the mask can reduce the occlusion problem between different layers. Otherwise, weighted complex loss function is implemented in the gradient descent optimization process, which analyzes the real part, the imaginary part, and the amplitude part of the focus region between the reconstructed images of the hologram and the target images. The weight parameter is used to adjust the ratio of the amplitude loss of the focus region in the whole loss function. The weight amplitude loss part in weighted complex loss function can decrease the interference of the focus region from the defocus region. The simulations and experiments have validated the effectiveness of the proposed method.

## 1. Introduction

The holographic three-dimensional (3D) display is considered one of the most ideal and promising 3D display technologies since it can reconstruct the whole optical wave field of the 3D scene. With the developments of computing technology, computer-generated holograms (CGHs) can be used to perform reconstruction of a 3D scene [1,2,3,4,5]. Double-phase holograms (DPHs) [6,7], the Gerchberg–Saxton (GS) algorithm [8], and its extended forms [9,10] are classic CGHs methods to generate the hologram. In recent years, optimizing the phase-only hologram by the stochastic gradient descent (SGD) method has been proposed [11,12,13,14] and developed rapidly.

In the process of SGD, the initial random phase is constantly updated as the loss value converges by iterating. The optimization process can approach the global minimum point of the loss function and obtain a high-quality hologram [15]. A traditional SGD method to generate multi-depth holograms is to compare the amplitude loss values between the reconstructed images of the hologram and the primitives at each depth and add the loss values together as a total loss. However, because there are no phase constraints in the optimization process, the phase distributions in the simulation reconstructed results are nonuniform. In addition, due to the non-ideal optical reconstruction system, this nonuniform phase distribution status will become worse; therefore, the optical reconstructed images are not consistent with simulation results, which are contaminated by speckle noise. Chen et al. [14] proposed a complex loss function based on the SGD method. Complex loss function can optimize both the amplitude term and phase term of the hologram. The phase distribution in the reconstructed results is nearly uniform and the speckle noise in the reconstructed results can be suppressed well. To use complex loss function, multiple images in different depths reconstruct each input image as the target planes at the corresponding depth [16,17]. The wave propagation of images in multi-depth to the target plane can be simulated by Fresnel diffraction or angular spectrum [18,19]. In the wave propagation process of multiple layers in different depths, the layers behind will gradually diffuse as the wave propagation and the occlusion will occur at the boundary of the previous layer. This occlusion problem will aggravate as the number of layers increases, especially when the contents of the input images are complex. Zhang et al. [20] adopted a mask to solve this occlusion problem. The mask is implemented in the process of forward propagation layer by layer and the usage of the mask leads to an outstanding display effect. Inspired by this method, we design a mask to solve occlusion problems between different layers. Whether it is forward wave propagation or backward wave propagation, the mask can decrease the interference between different layers. Otherwise, we add the amplitude loss of the focus region to complex loss function and adopt a weight parameter to adjust the ratio, which can reduce the interference of the focus region from the defocus region and promote the attention of observers to the focus region.

In this paper, the SGD method with weighted complex loss function and masked multi-layer diffraction is proposed to generate high-quality holograms of a 3D scene with multi-depth. Firstly, input image layers in different depths will diffract from the corresponding depth to the target plane. In the process of diffraction, a mask is used to solve the occlusion problem. By repeating the first step, we can get different target planes in different depths. Then, by comparing the real part, the imaginary part, and the amplitude part of the focus region of the holographic reconstructed images with that of the target planes at each depth and then adding them together as weighted complex loss function, the initial hologram can be updated constantly as the optimization process is close to the global minimum point of the loss function. Because of the usage of weighted complex loss function, the amplitude and the phase information of the holographic reconstructed images will be optimized simultaneously. Moreover, the weight parameter is used to adjust the ratio of the amplitude loss of the focus region in the whole loss function, and the amplitude loss part of the focus region can decrease the interference of the focus region from the defocus region. The proposed method can improve the quality of the hologram of the 3D scene with multi-depth.

## 2. Methods

**Figure 1:**
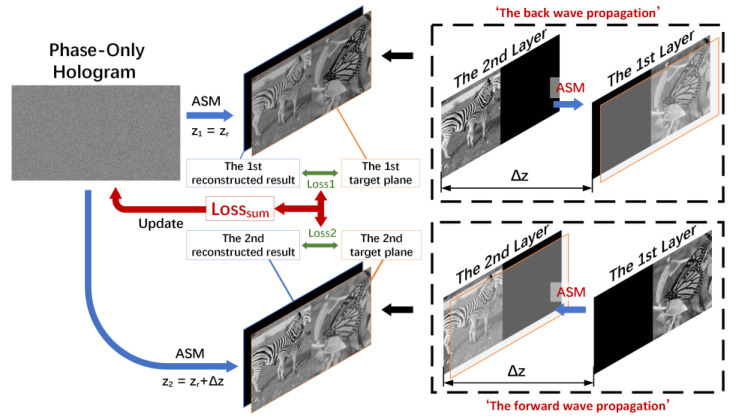
The proposed method.

The diagram of the proposed method is shown in Figure 1. Firstly, we adopt the target planes formed by layers diffracting in different depths as the primitives at each depth. Through the back and forward wave propagation of different layers, we can obtain the target planes at corresponding depths. The distance between adjacent layers is Δz and the mask is used to solve the occlusion problem in the diffracting process of layers. Then, the hologram is reconstructed in the different depths ($z_{1}=z_{r}$, $z_{2}=z_{r}+\Delta z$). The sum of the real loss, the imaginary loss, and the amplitude loss of the focus region between the target planes and the reconstructed images are implemented as weighted complex loss function. We use weighted complex loss function for the SGD method updating the initial random phase in the process of the SGD. After many iterations of the SGD method, we can get a high-quality multi-depth hologram.

We take the forming process of the 1st target plane as an example. The 2nd layer propagates back to the 1st layer to form the 1st target plane. The angular spectrum method (ASM) is used to simulate wave propagation. The complex amplitude of the ith layer after wave propagation is given by:

(1) $$U_{i}(x,y)=IFFT\{FFT[A_{i}(x,y)]\times H_{i}\},$$

where $A_{i}\left(x,y\right)$ is the amplitude of the ith layer, FFT represents the fast Fourier transform operator, and IFFT represents inverse FFT. $H_{i}$ represents the transform function in ASM. The expression of $H_{i}$ is expressed by the following equation:

(2) $$H_{i}=\left\{\begin{array}{l} e^{i\frac{2\pi }{\lambda }z_{i}}\sqrt{1-{\left(\lambda f_{x}\right)}^{2}-{\left(\lambda f_{y}\right)}^{2}},if\sqrt{f_{x}{}^{2}+f_{y}{}^{2}}<\frac{1}{\lambda }\\ 0\;\;\;\;\;\;\;\;\;\;\;\;otherwise \end{array}\right.,$$

where $z_{i}$ is the diffraction distance, λ is the wavelength, and $f_{x}$, $f_{y}$ are the spatial frequencies in the x and y dimensions. To solve the occlusion problem in the diffracting process, the complex amplitude of the focus layer (the 1st layer) after wave propagation needs to multiply a mask:

(3) $$M(m,n)=\left\{\begin{array}{l} 0,\;\;\;0<m<960,0<n<1080\\ 1,\;\;\;960<m<1920,0<n<1080 \end{array}\right.,$$

where *m* and *n* are the numbers of sampling points of the object in the x and y dimensions, respectively. By setting the mask with a binary value, it is effective to pass the wavefront where the region of 960<m<1920, 0<n<1080 and let the rest block. Then, the complex amplitude of the 2nd layer (the defocus layer) $U_{2}\left(x,y\right)$ is added to the product of the mask and the complex amplitude of the 1st layer (the focus layer) $U_{1}\left(x,y\right)$, which forms the complex amplitude of the 1st target plane:

(4) $$C_{1}(x,y)=U_{1}(x,y)\times M(m,n)+U_{2}(x,y),$$

Repeating the analogous operation of the forming process of the 1st target plane, the 2nd target plane can be obtained. However, unlike the formation process of the 1st target plane, the formation process of the 2nd target plane is the forward propagation between layers.

We adopt a random phase $p_{initial}$ in the first iteration of the SGD as the hologram. In the process of SGD, the import phase updated by the previous iteration is reconstructed by ASM, which is described as:

(5) $$R_{i}=IFFT\{FFT[h]\times H_{r}\},$$

where $R_{i}$ is the complex amplitude of the reconstructed result of the hologram in different depths, h represents the wavefront of $p_{initial}$, which is expressed as $h=e^{ip_{initial}}$. $H_{r}$ represents the transform function in the process of reconstruction, the expression of which is:

(6) $$H_{r}=\left\{\begin{array}{l} e^{-i\frac{2\pi }{\lambda }z_{r}}\sqrt{1-{\left(\lambda f_{x}\right)}^{2}-{\left(\lambda f_{y}\right)}^{2}},\;\;if\sqrt{f_{x}{}^{2}+f_{y}{}^{2}}<\frac{1}{\lambda }\\ 0\;\;\;\;\;\;\;\;\;\;\;\;\;otherwise \end{array}\right.,$$

where $z_{r}$ is the diffraction distance between the hologram and the jth target plane, λ is the wavelength, and $f_{x}$, $f_{y}$ are the spatial frequencies in the x and y dimensions.

The loss function plays an important role in the SGD optimization process. Images in different depths will focus at the corresponding depth while the other image will be blurry, so the focus region needs more attention than the defocus region [21]. In the proposed method, the sum of the real loss, the imaginary loss, and the amplitude loss of the focus region between the reconstructed images of the hologram and the target planes are implemented as weighted complex loss function, which is given by:

(7) $$Loss_{sum}=\sum_{j=1}^{n}MSEloss(R_{r},R_{t})+MSEloss(I_{r},I_{t})+m\times MSEloss(A_{r}\times M_{j},A_{j}\times M_{j}),$$

where $Loss_{sum}$ is the loss sum, *n* is the number of the planes, $R_{r}$, $I_{r}$ and $A_{r}$ are the real part, the imaginary part, and the amplitude of the holographic reconstructed image, $R_{t}$, $I_{t}$ and $A_{j}$ are the real part, imaginary part, and the amplitude of the jth target plane, the parameter m represents the weight, and $M_{j}$ represents a binary mask highlighting the focus region of the holographic reconstructed results and the target planes. *MSEloss* is the mean squared error, which can be expressed as:

(8) $$MSEloss=\frac{1}{mn}{\sum_{m,n}\left[S_{r}(m,n)-S_{t}(m,n)\right]}^{2},$$

where *S_r_* and *S_t_* represent the results of the holographic reconstructed image and the target planes, respectively.

The amplitude loss part of the focus region in weighted loss function can make the boundary between the focused region and the defocused region clearer. By setting m and $M_{j}$, it can promote the attention of observers to the focus region and control the display effect of the focus region and the defocus region.

The SGD method can optimize the initial phase in succession based on weighted complex loss function by iterating. Finally, the hologram of the 3D scene and its reconstructed image can be obtained. The mask can solve the occlusion problem between different layers in the process of the images diffracting in different depths. In addition, weighted complex loss function can improve the quality of the hologram and make the edge between the focus region and the defocus region clear.

## 3. Results

### 3.1. Simulation Results

**Figure 2:**
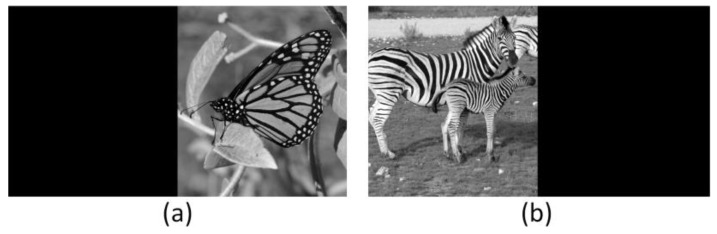
(**a**) ‘butterfly’ and (**b**) ‘zebra’ are used to represent 2 different depth layers of the 3D scene.

To verify the feasibility of the method, we performed relevant experiments. Figure 2a ‘butterfly’ and (b) ‘zebra’ from the DIV2K dataset are used to represent two different depth layers of the 3D scene in the experiments. The resolutions of ‘butterfly’ and ‘zebra’ including the black areas are all 1080 × 1920 pixels. The distance $z_{r}$ between the hologram and the first target plane is 230 mm. To observe the influence of the occlusion effect when the distance between adjacent layers is different, we set the distance between adjacent layers Δz to 10 mm, 30 mm, and 50 mm, respectively. The spatial light modulator (SLM) has 1080 × 1920 pixels, the pixel pitch is 8 μm, and the wavelength of the light source is 639.0 nm. During simulations, the initial phase $p_{initial}$ is supposed to be a random phase with a range of (−π, π). In a large number of simulation experiments, we find that the display effect between the focus region and the defocus region will achieve an appropriate balance when the weight parameter is between 4.5 and 5. Between 4.5 and 5, we adopt 4.8 as the weight parameter m in weighted complex loss function. The number of the iteration in the process of SGD is set to 100 and the learning rate of SGD is set to 0.1. Pytorch 1.10.0 and Python 3.8.0 are implemented to optimize the process of SGD. The Adam optimizer is used to update the learning rate in the optimization process.

**Figure 3:**
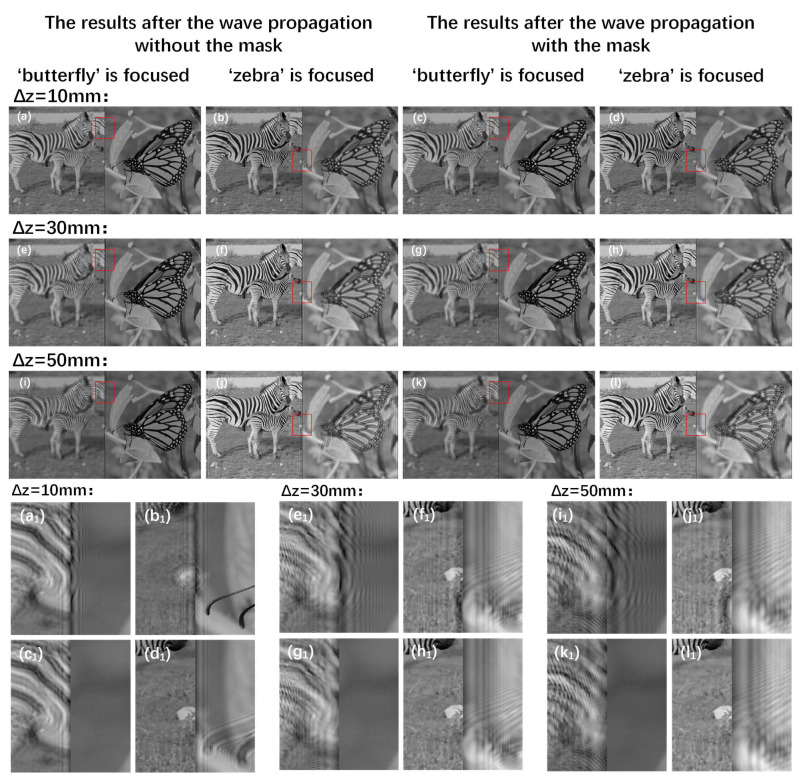
(**a**,**b**), (**e**,**f**), and (**i**,**j**) are the results after propagation without the mask when the distance Δz is 10 mm, 30 mm, and 50 mm, respectively. (**c**,**d**), (**g**,**h**), and (**k**,**l**) are the results after propagation with the mask. (**a_1_**–**d_1_**), (**e_1_**–**h_1_**), and (**i_1_**–**l_1_**) are the details of (**a**–**d**), (**e**–**h**), and (**i**–**l**) in the red box.

Figure 3 shows the results (the first target plane and the second target plane) after the wave propagation of layers in different depths without the mask and with the mask when the distance Δz is 10 mm, 30 mm, and 50 mm, respectively. As shown in Figure 3a_1_–l_1_, the occlusion problem of the wave propagation without the mask is aggravated according to the increase of the propagation distance. The usage of the mask can solve this occlusion problem well.

**Figure 4:**
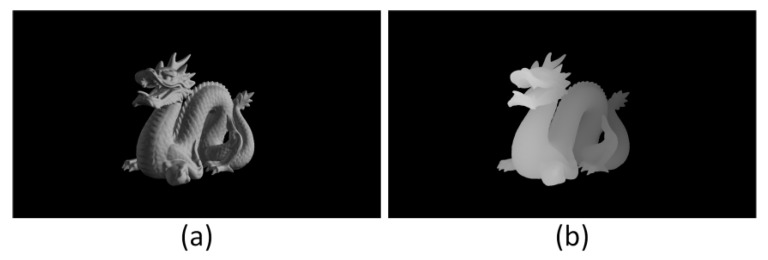
(**a**) ‘dragon’ and (**b**) its depth map are used to represent 2 different depth layers of the 3D scene.

Figure 3 shows the simple vertical occlusion case. Otherwise, we use the mask in a complex situation to verify the validity of the mask. As shown in Figure 4, we use ‘dragon’ and its depth map to simulate complex diffracting processes.

**Figure 5:**
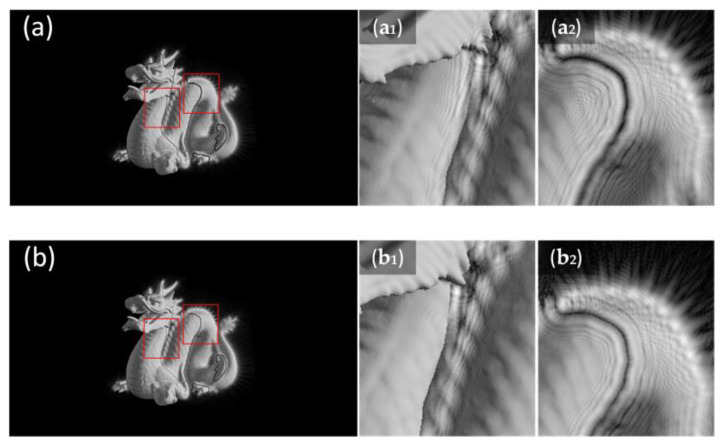
(**a**,**b**) are the results after the wave propagation without the mask and with the mask when Δz is 10 mm. (**a_1_**,**b_1_**) and (**a_2_**,**b_2_**) are the details of (**a**,**b**) in the red box region, respectively.

By setting the depth values in different ranges, we can obtain different layers. After the wave propagation of layers, we can get the target plane. Figure 5 is the comparison of the results after the wave propagation of layers generated by the ASM without the mask and with the mask. We can see that the edge between different layers after the wave propagation with the mask is clearer. The mask is still effective while handling irregular graphics.

**Figure 6:**
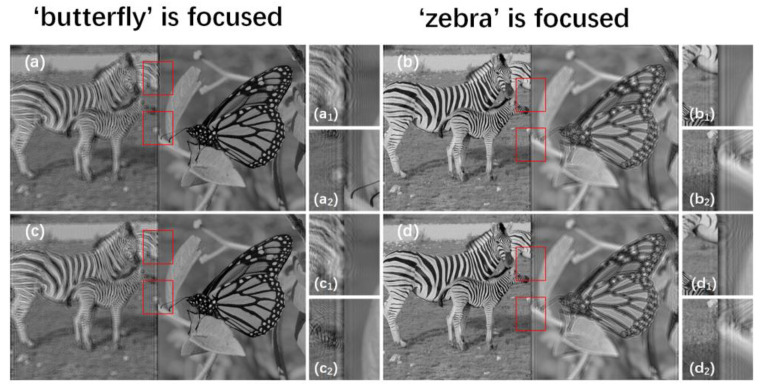
(**a**,**b**) are the reconstructed results of the SGD method with complex loss function and (**c**,**d**) are the reconstructed results of the proposed method when the distance $z_{r}$ is 230 mm and Δz is 30 mm. (**a_1_**–**d_1_**) and (**a_2_**–**d_2_**) are the details of (**a**–**d**) in the red box region, respectively.

After obtaining the first target plane and the second target plane, we use the SGD method with weighted complex loss function to update the initial phase and obtain high-quality holographic reconstructed images. Because the amplitude loss part of the focus region is used in the whole loss function, the focus regions of the reconstructed images acquire more attention than the defocus regions and obtain higher quality. The comparisons of the reconstructed results between the SGD method with complex loss function and the proposed method when $z_{r}\;$= 230 mm and Δz is 30 mm are shown in Figure 6. The interference between the focus regions and the defocus regions of the proposed method is slighter than that of the SGD method with complex loss function.

**Figure 7:**
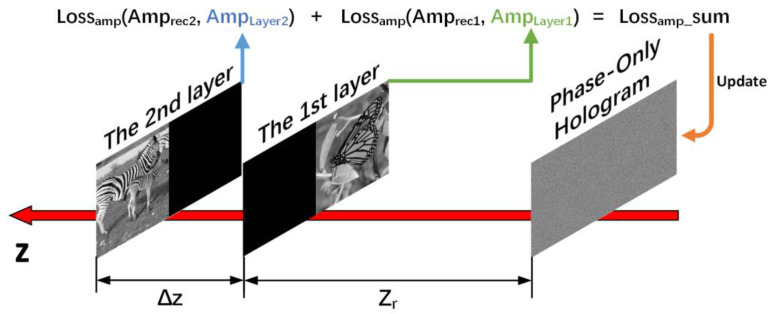
The SGD method with amplitude loss function generates multi-depth holograms.

Figure 7 shows the SGD method with amplitude loss function to generate multi-depth holograms. The SGD method with amplitude loss function only calculates the amplitude loss values between the reconstructed images of the hologram and the primitives at each depth.

**Figure 8:**
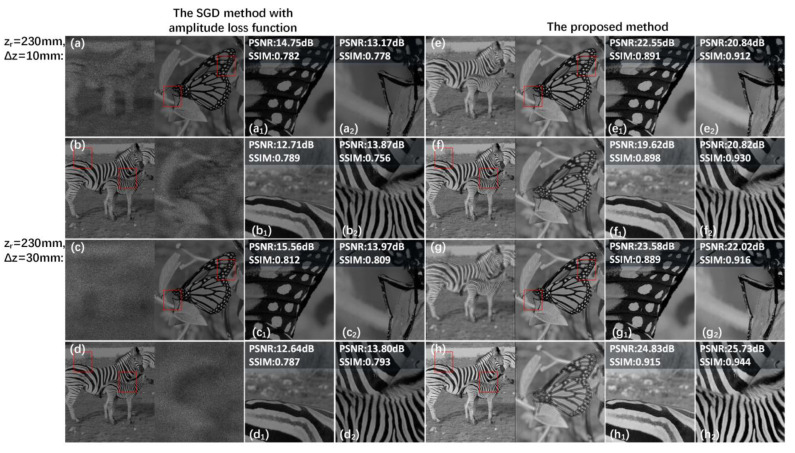
(**a**–**d**) are the reconstructed results of the SGD method with amplitude loss function and (**e**–**h**) are the reconstructed results of the proposed method when the distance $z_{r}$ is 230 mm and Δz is 10 mm and 30 mm, respectively. (**a_1_**–**h_1_**) and (**a_2_**–**h_2_**) are details of (**a**–**h**) in the red box region, respectively.

The comparisons of reconstructed results among the SGD method with amplitude loss function and the proposed method are given in Figure 8. Figure 8a–d gives the reconstructed results of the SGD method with amplitude loss function. Because of only calculating the amplitude loss values between the reconstructed images of the hologram and the primitives at each depth, there are no phase constraints in the optimization process, so the reconstructed image quality both of the focus regions and the defocus regions is unsatisfactory. As shown in Figure 8e–h, the reconstructed results generated by the proposed method are of high quality, and the focus regions of the reconstructed images almost do not contain the interference from the defocus regions. (a_1_–h_1_) and (a_2_–h_2_) are the details of the previously reconstructed images in the red box region. The peak signal-to-noise ratio (PSNR) values and structural similarity (SSIM) values in the red box region of the proposed method are all higher than that of the SGD method with amplitude loss function, which shows that the quality and display effect of the reconstructed images in different depths generated by the proposed method are all better.

As shown in Table 1, we compared the time consumed between the SGD method with complex loss function and the proposed method. Because the loss function of the proposed method is calculated at each target plane, the proposed method does consume more time than the SGD method with complex loss function. It is indeed a limitation of the proposed method.

### 3.2. Optical Results

**Figure 9:**
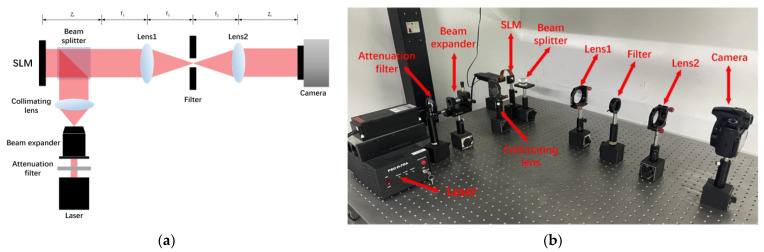
(**a**) is the Schematic of the optical setup. (**b**) shows all the devices needed for the optical experiment.

The optical setup is shown in Figure 9. The 4-*f* filter system is used in the optical reconstruction process of the hologram, and the focal lengths of the two lenses (lens 1 and lens 2) are both 150.0 mm. A phase-only SLM (8 µm pixel pitch, 1080 × 1920 pixels) is used, which is provided by HOLOEYE company. The frame rate and the phase modulation range of the SLM are 60 Hz and [0, 2π], respectively. The wavelength of the input red light laser is 639.0 nm. The hologram is displayed on the SLM and the reconstructed results were captured by a complementary metal oxide semiconductor (CMOS) camera (Canon 60D).

**Figure 10:**
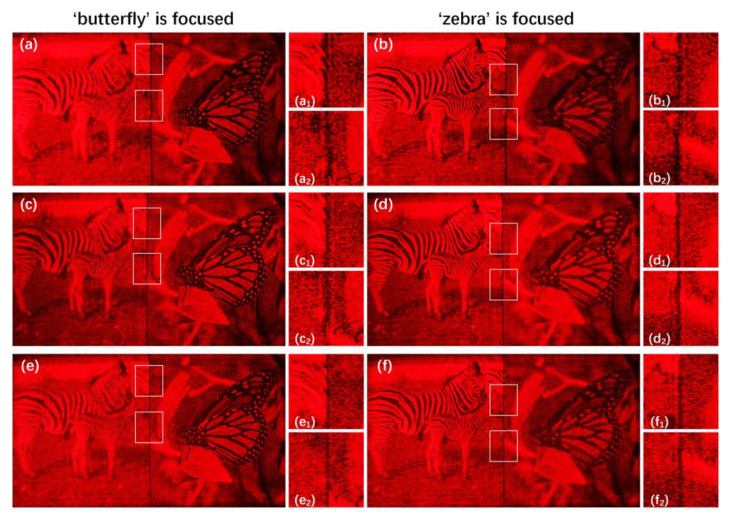
(**a**,**b**) are the optical reconstructed results of the SGD method with weighted complex loss function when the mask is not used in the process of the diffraction, (**c**,**d**) are the optical reconstructed results of the SGD method with complex loss function when the mask is used in the process of the diffraction, and (**e**,**f**) are the optical reconstructed results of the proposed method when the distance $z_{r}$ is 230 mm and Δz is 30 mm. (**a_1_**–**f_1_**) and (**a_2_**–**f_2_**) are details of (**a**–**f**) in the white box.

Figure 10a,b shows the optical reconstructed results of the SGD method with weighted complex loss function when the mask is not used in the process of the diffraction. Because the mask is not used, the results contain serious interference between different layers. Figure 10c,d shows the optical reconstructed results of the SGD method with complex loss function when the mask is used in the process of diffraction. The aliasing between different layers is slighter than the former method. Figure 10e,f gives the optical reconstructed results of the proposed method. Because of the usage of the mask and weighted complex loss function, the interference between the focus regions and the defocus regions of the proposed method is the slightest among these three methods, and the image edge are also the clearest.

**Figure 11:**
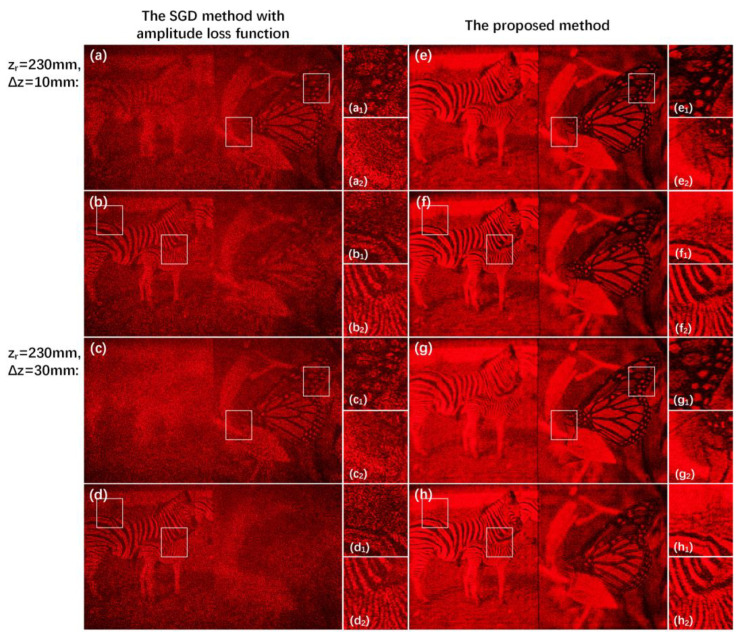
(**a**–**d**) are the optical reconstructed results of the SGD method with amplitude loss function and (**e**–**h**) are the optical reconstructed results of the proposed method when the distance $z_{r}$ is 230 mm and Δz is 10 mm and 30 mm, respectively. (**a_1_**–**h_1_**) and (**a_2_**–**h_2_**) are the details of (**a**–**h**) in the white box, respectively.

The comparisons of the optical reconstructed results between the SGD method with amplitude loss function and the proposed method are given in Figure 11. As shown in Figure 11a–d, the optical reconstructed results of the SGD method with amplitude loss function are not consistent with the simulation results, which are still contaminated by speckle noises because the optical reconstruction system is not an ideal system and it aggravates the nonuniform status of the phase distribution. The optical reconstructed images of the proposed method are shown in Figure 11e–h, and their display effect is more excellent than that of the SGD method with amplitude loss function. Compared with the SGD method with amplitude loss function, the optical reconstructed images generated by the proposed method agree well with the simulation results, and the speckle noise is suppressed well. Because the proposed method optimizes phase and amplitude simultaneously, the phase distribution of the optical reconstructed image is more uniform than that of the SGD method with amplitude loss function. Therefore, the influence from the error of a nearly uniform phase distribution is smaller than a nonuniform phase distribution even in a non-ideal optical reconstruction system.

## 4. Conclusions

In conclusion, we propose a new SGD method to generate a high-quality hologram of a 3D scene with multi-depth. The mask decreases the interference between different layers in the diffracting process of different layers, which ensures the focus region is not contaminated by the defocus region. Weighted complex loss function can improve the quality and control the display effect of the focus region and the defocus region of the hologram.

## Figures and Tables

**Table 1:** Comparison of the optimization time for 100 steps.

Method	$z_{r}\;$ = 230 mm, Δz = 10 mm	$z_{r\;}$= 230 mm, Δz = 30 mm	$z_{r}\;$= 230 mm, Δz = 50 mm
The proposed method	48.9338 s	47.5858 s	48.8018 s
The SGD method with complex loss function	30.6387 s	28.7206 s	28.1467 s

## Data Availability

Not applicable.
